# Outcome after Partial Pulpotomy: Long-term Results of the Prospective Clinical MMP-9 Study

**DOI:** 10.3290/j.ohpd.b4438901

**Published:** 2023-09-27

**Authors:** Jelena Petrovic, Caroline Sekundo, Holger Gehrig, Sarah Rampf, Shirin El-Sayed, Alexander Ritz, Johannes Mente

**Affiliations:** a Dentist and Lecturer, Department of Conservative Dentistry, Division of Endodontics and Dental Traumatology, University Hospital Heidelberg, Germany. Conceptualisation, data curation, formal analysis, investigation, validation, wrote original draft of the manuscript, reviewed and edited the manuscript.; b Dentist and Lecturer, Department of Conservative Dentistry, Division of Preventive and Restorative Dentistry, University Hospital Heidelberg, Germany. Visualisation, formal analysis, validation, supervision, wrote and edited the manuscript.; c Dentist, Department of Conservative Dentistry, Division of Endodontics and Dental Traumatology, University Hospital Heidelberg, Germany. Conceptualisation, methodology, investigation, data curation, supervision, wrote, reviewed and edited the manuscript.; d Dentist and Lecturer, Department of Conservative Dentistry, Division of Endodontics and Dental Traumatology, University Hospital Heidelberg, Germany. Conceptualisation, methodology, investigation, wrote, reviewed and edited the manuscript.; e Dentist and Lecturer, Department of Conservative Dentistry, Division of Endodontics and Dental Traumatology, University Hospital Heidelberg, Germany. Investigation, data curation, validation, wrote, reviewed and edited the manuscript.; f Statistician, Faculty of Mathematics/Computer Science and Mechanical Engineering, Technische Universität Clausthal, Clausthal-Zellerfeld, Germany. Formal analysis, supervision, wrote, reviewed and edited the manuscript.; g Professor, Department of Conservative Dentistry, Division of Endodontics and Dental Traumatology, University Hospital Heidelberg, Germany. Conceptualisation, methodology, data curation, investigation, project administration, supervision, validation, wrote original draft of the manuscript, reviewed and edited manuscript.

**Keywords:** carious pulp exposure, mineral trioxide aggregate, MMP-9, outcome, partial pulpotomy, vital pulp therapy

## Abstract

**Purpose:**

While the objective of partial pulpotomy is to preserve the vitality and function of the pulp tissue, the preoperative pulp status is the main prognostic factor for its success. To date, however, there is little data on long-term success rates. Therefore, the aim of this prospective pilot study was to assess the long-term outcome of partial pulpotomy in permanent teeth after carious pulp exposure without signs or symptoms of irreversible pulpitis, verified clinically, radiographically, and via MMP-9 levels.

**Materials and Methods:**

Patients in whom permanent teeth with extremely deep carious lesions were diagnosed as completely asymptomatic (n = 8) or with signs of reversible pulpitis (n = 10) underwent non-selective caries removal followed by a blood test to assess the level of MMP-9. The teeth were thereafter partially pulpotomised, MTA-capped, and immediately restored with composite resin. Follow-up examinations were performed by endodontically experienced examiners focusing on clinical and radiographic assessment.

**Results:**

One patient could not be contacted and was lost to follow-up. Overall, the follow-up period ranged from 2–8 years (mean = 4.4 years). The majority of teeth remained functional and without pathology; one tooth was classified as having failed because of a vertical root fracture. There was no statistically significant difference in the groups’ success rate (p = 0.3). The estimated overall survival rate was 94.1% (95% CI: 0.84-1.00) after 4 years according to the Kaplan-Meier method.

**Conclusion:**

Pulp vitality in permanent teeth can be preserved with high success rates by means of partial pulpotomy after carious pulp exposure in asymptomatic teeth or in teeth with reversible pulpitis.

Vital pulp therapy (VPT), such as pulp capping, partial or full pulpotomy, consists of a set of measures intended to preserve the vitality and function of the pulp tissue. Recently, different expert groups issued position statements about VPT, including diagnostic and therapeutic recommendations.^1,15^ The main diagnostic consideration emphasised therein is the importance of the pulp’s preoperative inflammatory state for the outcome of VPT. This is supported by a recent systematic review, identifying the preoperative pulp status as the only significant prognostic factor for the success rate of partial pulpotomy.^13^ The assessment of the inflammatory state of the pulp should therefore be evaluated as precisely as possible before VPT. Established clinical parameters include the patient’s pain history, thermal and electric pulp testing (EPT), periapical radiographic examination, and the response to percussion. Additionally, the measurement of inflammatory mediators such as matrix metalloproteinases (e.g., MMP-9) has been proposed in recent years.^5,27,35,45^ Although commercially available molecular chairside tests for everyday clinical use are still unavailable, the use of MMP-9 as a predictive local biomarker may help assess the infiltration and breakdown of the dental pulp tissue and thus the outcome of VPT.^5^

In regard to the available treatment strategies, the results of clinical studies indicate that partial pulpotomy, with success rates of > 92% after ≥ 2 years of follow-up,^2,13,21^ results in a more favourable outcome than direct pulp capping, with a weighted pooled success rate of 87.7% at > 2-3 years and 72.9% at > 3 years.^2,21^ To date, only a few studies – predominantly evaluated in young permanent teeth – have assessed long-term outcomes of partial pulpotomy.^8,23,25^ However, data from well-designed long-term trials is required.^1,21^ Moreover, to the best of our knowledge, no study has yet assessed long-term survival outcomes in teeth whose preoperative inflammatory pulp status was verified clinically, radiographically, and through the measurement of inflammatory mediators.

For this reason, the present study aimed to assess the long-term outcome of partial pulpotomy in permanent teeth after carious pulp exposure without signs or symptoms of irreversible pulpitis. The inflammatory state of the pulp of all teeth was verified clinically, radiographically and via MMP-9 levels.^27^

## Materials and Methods

This prospective, two-centre clinical study was performed among patients recruited from the Department of Conservative Dentistry of the University Hospital of Heidelberg, Germany. and a private dental practice located nearby. The study protocol was approved by the Ethics Committee of the University of Heidelberg (Ref. S-219/2012). Informed written consent was obtained from all study participants.

Patients aged 12 years or older, who presented with a permanent tooth exhibiting an extremely deep carious lesion, were included in the study. The term ‘extremely deep carious lesion’ refers to caries that has penetrated through the entire dentin thickness, making pulp exposure inevitable during the excavation process.^15^ Teeth which were diagnosed as completely asymptomatic before treatment (no clinical signs of pulpitis, no history of pain, response to cold testing within normal limits, no sensitivity to percussion or bite testing, bleeding time from the exposed pulp tissue < 2 min, periodontally healthy, periapical index [PAI] = 1)^30,44^ or with reversible pulpitis (slightly exaggerated reaction to cold or sweet stimuli, no history of spontaneous pain, no sensitivity to percussion or bite testing, bleeding time from the exposed pulp tissue <5 min, periodontally healthy, PAI = 1) were included in this analysis. All teeth underwent partial pulpotomy followed by pulp capping using mineral trioxide aggregate (MTA). Blood samples were obtained from the dental pulp for the measurement of MMP-9 levels.

Teeth that were extensively damaged and could not undergo treatment under rubber-dam isolation were not included in the study. Patients with compromised immune status, those who were pregnant or had used medications that could affect MMP-9 levels (such as antibiotics, bisphosphonates, statins or NSAIDs) within the four weeks preceding the intervention were excluded. For further information on other study groups, and detailed results on MMP-9 levels, please see our previous publication.^27^

### Treatment Intervention

The treatment procedure, blood sample collection and recording of clinical findings were standardised as much as possible with the aid of predefined study and treatment protocols. All investigators at the university hospital and the cooperating private practice were instructed accordingly by the principal investigator (J.M.).

Before and after treatment, a digital radiograph (VistaScan PSP System, Dürr Dental; Bissingen, Germany) was taken, using the paralleling technique. All teeth were anaesthetised using articain with epinephrine (1:200 000, Ultracain D-S, Sanofi-Aventis; Frankfurt am Main, Germany) and treated under rubber-dam isolation. After non-selective caries removal, partial pulpotomy was performed by an endodontically experienced study investigator using magnifying loupes or a dental operating microscope. Caries excavation was performed from the peripheral to the central, until the pulp was exposed, using sterile high-speed diamond burs under constant water cooling and slow-speed rose-head burs (Brasseler; Lemgo, Germany). The size of the exposed pulp was measured using a millimeter-scaled periodontal probe (PCPUNC15, Hu-Friedy; Chicago, IL, USA). Partial pulpotomy was performed using sterile high-speed diamond burs under constant water cooling. Next, blood samples were collected from the exposed pulp using heparinised 10-ml microcapillary tubes (Hirschmann; Eberstadt, Germany) for the measurement of MMP-9 levels. The cavity was then disinfected using sterile cotton pellets soaked with 0.12% chlorhexidine solution (Glaxo Smith Kline; Bühl, Germany) until hemostasis was achieved. Bleeding duration was recorded. Afterwards, the pulpal wound was dressed with ProRoot MTA white (Dentsply Maillefer; Ballaigues, Switzerland). The teeth were immediately restored with composite resin (Tetric EvoCeram, Vivadent; Schaan, Liechtenstein). The blood samples were transported in a cool box at a temperature of -25°C to the laboratory where they were prepared for evaluation of MMP-9 levels (for details, see previous publication).^27^

### Follow-up Examinations

Follow-up examinations were carried out annually by three endodontically experienced clinicians (J.P., S.E. and S.R.). Special attention was given to the clinical and radiographic examination of the treated teeth. The follow-up procedure consisted of: 1. questions with regard to any history of pain or discomfort or dental trauma post-treatment; 2. clinical examination of the treated tooth focusing on the response to cold testing (refrigerant spray), tenderness to percussion, presence of a sinus tract, probing depths, attachment loss, tooth mobility, furcation involvement, type and quality of restoration, secondary caries, or clinical signs of a root fracture.

### Radiographic Assessment

All dental radiographs were taken with an RWT film holder (KKD; Ellwangen, Germany) and a photostimulable phosphor image plate system (VistaScan PSP System, Dürr Dental) using the paralleling technique. These were subsequently evaluated in a darkened room utilising a special computer screen (21.3-inch, EIZO RadiForce R22, EIZO; Rülzheim, Germany; resolution: 1600 x 1200 pixels; pixel pitch: 0.270 x 0.270 mm; contrast ratio: 550:1).

The radiographic evaluation of the treated tooth was performed with attention to pathological changes, such as signs of root resorption, obliteration, caries, root fracture, integrity of the restoration, formation of a hard tissue barrier below the pulp-capping material, and the periapical status using the PAI.^30^ Radiographic assessment was undertaken by two experienced examiners (J.P., H.G.) who also determined the PAI. In cases of disagreement, a third endodontist (J.M.) also performed the assessment and a consensus was reached. Multirooted teeth were assessed according to the highest-scored root on the PAI.

Prior to evaluating the study radiographs, the examiners were calibrated with the PAI calibration kit of 100 periapical radiographs.^30^ Intra-examiner reliability and inter-examiner agreement with the calibration kit’s ‘gold standard’ were assessed using Cohen’s Kappa.

### Outcome Measures

The primary outcome was the success of the partial pulpotomy. The outcome was considered a success if all of the following findings were observed in the treated tooth at all follow-up examinations: absence of clinical signs and symptoms, no history of pain or discomfort persisting longer than 3 months post-treatment, response to cold testing within normal limits, and no sensitivity to percussion or bite testing. In regard to the radiographic examination, the following findings were required to classify the tooth as a success: no indication of apical periodontitis (PAI score = 1), no widening of the periodontal ligament space, no condensing osteitis, no loss of function (e.g., grade III tooth mobility), no root fracture, and the absence of internal or external root resorption. The outcome was classified as a failure if any of the above criteria were not met.

### Statistical Analysis

The Cohen’s Kappa test was used for PAI calibration, following the recommendations described by Ørstavik et al.^30^ Exploratory analyses were performed by descriptive means, calculating mean (±SD), median, first and third quartiles, minima and maxima for continuous variables, as well as relative and absolute frequencies for categorical variables. Survival times (i.e., time from treatment until the date of last contact or ‘failure’) were calculated using the Kaplan-Meier estimation. Statistical analyses were conducted using R (Version 4.1.2, http://www.r-project.org/).

## Results

### Intra- and Inter-examiner Calibration

In the scope of PAI calibration, Cohen’s weighted kappa values for intra-examiner reliability were κ = 0.83 (J.P.) and κ = 0.81 (H.G.), and for inter-examiner agreement (examiner scores vs the calibration kit’s ‘authorized score’) were κ = 0.94 for J.P. and κ =0.88 for H.G. All four kappa values indicate an almost perfect agreement.^20^

### Study Cohort

The baseline characteristics of the study population are summarised in Table 1. Eighteen patients, with one treated tooth each, were included in the analysis. All teeth were treated by means of partial pulpotomy, followed by MTA pulp dressing; eight of the teeth were asymptomatic before treatment, and ten were diagnosed with reversible pulpitis. The first follow-up examination took place approximately one year after treatment. One patient could not be contacted despite repeated attempts and was completely lost to follow-up. In total, follow-up ranged from 2-8 years, with a mean follow-up period of 4.4 (± 2.1) years (median 4 years). The overall patient recall rate was 94%.

**Table 1:** Outcome distribution across pre-, intra- and postoperative variables for both diagnosis related groups of teeth (asymptomatic teeth and those with reversible pulpitis) treated with partial pulpotomy

Variable			Diagnosis				Total		p-value £	OR (95% CI)
			Asymptomatic tooth		Reversible pulpitis					
			n (%)		n (%)		n (%)			
Age (years)		Mean (SD)	29.0 (7.17)	8 (100)	42.49 (21.21)	10 (100)	36.5 (17.51)	18 (100)	0.24¥	1.007# (0.99-1.21)
		Median	27.96		34.15		29.03			
		Min-Max	19-42		20-74		19-74			
Sex	Male			7 (88)		3 (30)		10 (56)	0.017†	0.061 (0.01-0.74)
	Female			1 (12)		7 (70)		8 (44)		16.33 (1.35-197.77)
Tooth type	Anterior			1 (12)		1 (10)		2 (11)	>0.99†	1.29 (0.068-24.38)
	Posterior			7 (88)		9 (90)		16 (89)		0.78 (0.04-14.75)
Tooth location	Maxilla			5 (62)		7 (70)		12 (67)	0.83†	1.4 (0.2-10.03)
	Mandible			3 (38)		3 (30)		6 (33)		0.71 (0.1-5.12)
Size of exposed pulp (in mm2)		Mean (SD)	1.59 (0.71)		2.72 (1.43)		2.22 (1.27)		0.043¥	3.76# (1.14-23.71)
		Median	1.5		2.38		2.0			
		Min-Max	1-3		1-6		1-6			
Time of bleeding after partial pulpotomy	< 2 min			8 (100)		4 (40)		12 (67)	0.009†	NA*
	2-5 min			0 (0)		6 (60)		6 (33)		NA*
MMP-9 level (in ng/ml)		Mean (SD)	422.43 (168.06)	8 (100)	920.99 (380.35)	10 (100)	699.41 (391.41)	18 (100)	0.006¥	1.006# (1.0017-1.014)
		Median	385.75		938.54		574.84			
		Min-Max	267.54-807.16		300.25-1395.73		267.54-1395.73			
Type of postoperative restoration	Composite			7 (88)		7 (78)		14 (82)	0.47ƒ	NA*
	Crown and partial crown			0 (0)		2 (22)		2 (12)		NA*
	Inlay			1 (12)		0 (0)		1 (6)		NA*
Quality of postop. restoration‡	Acceptable			8 (100)		9 (90)		17 (94)§	NA	NA*
	Unacceptable			0 (0)		0 (0)				

¥ Mann-Whitney U-test; † Boschloo’s test; ƒ Fisher’s exact test; ₤ statistically significant at 5% confidence level; § one missing value, because 1 patient did not take part in follow-up; ‡ based on radiographic and clinical assessment; # exponentiated coefficient of the relevant variable within a logistic regression, i.e. odds ratio for a one unit increase in the variable; * the odds ratio according to Wald is not calculated for categorical variables with cell counts of zero; OR: odds ratio; CI: confidence interval; NA: no test; bold denotes statistical significance at a 5% confidence level.

The age of the study participants ranged between 19 and 74 years, with a median age of 28.5 years (mean 36.5 [±17.5] years; first quartile: 25.9 years, third quartile: 42.1 years). Ten (10) of the patients were male and 8 were female. There were considerably more women in the group diagnosed with reversible pulpitis (p = 0.02).

### Clinical and Radiographic Assessment

Intra- and postoperative characteristics are shown in Table 1. Preoperative radiographic assessment showed all teeth with a PAI = 1, and was therefore uneventful. No patient complained about post-operative pain. Two patients with previously asymptomatic teeth presented with slight post-treatment discomfort, which subsided after a few weeks (no longer than 8 weeks). At all follow-up examinations, the pulpotomised teeth were completely asymptomatic, the percussion tests were negative, the sensitivity tests with refrigerant spray were positive. Postoperative probing depths (mean 2.9 mm ± 1.5 mm; median 3 mm) were comparable to the preoperative probing depths (mean 2.9 mm ± 0.9 mm; median 3 mm), with the exception of one tooth, which had a probing depth of 8 mm at follow-up due to a longitudinal root fracture. There was no increased mobility or furcation involvement at any follow-up examination. Clinically and radiographically, all restorations showed adequate margins without any sign of secondary caries, and the PAI was unchanged (PAI = 1 in all cases).

**Fig 1:**
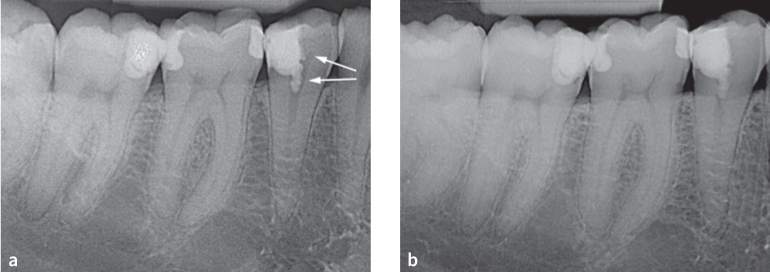
(a) Postoperative radiograph of a mandibular second premolar after partial pulpotomy, MTA capping, and immediate restoration with composite resin (arrows pointing to the clearly identifiable pulp horn). (b) Follow-up radiograph after 8 years. The pulp horn is no longer visible, but no obliteration of the root canal below the pulp capping material (MTA) is identifiable.

**Fig 2:**
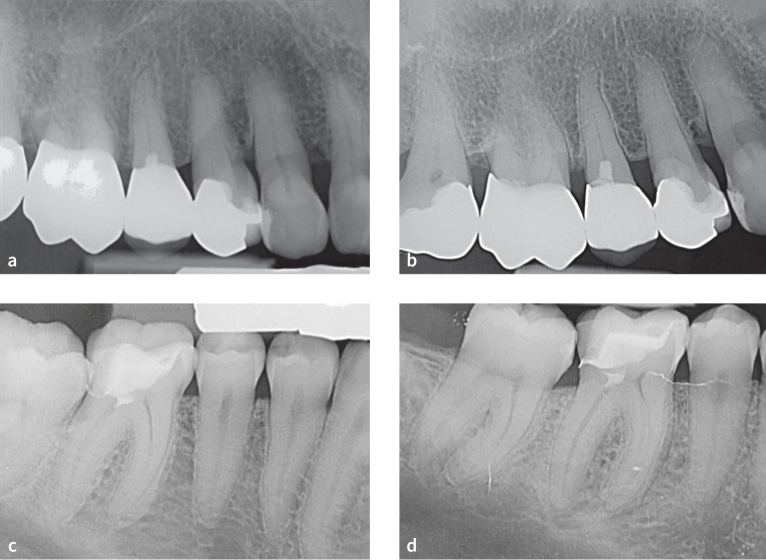
(a) Postoperative radiograph of a maxillary second premolar after partial pulpotomy, MTA-pulp capping, and immediate restoration with composite resin. (b) Follow-up radiograph of the tooth after 6 years. There is no evidence of obliteration of the root canal below the pulp capping material (MTA). (c) Postoperative radiograph of a mandibular first molar after partial pulpotomy, MTA-pulp capping, and immediate restoration with composite resin. (d) The follow-up radiograph of the tooth after 5.5 years showed no signs of obliteration of the root canal below the pulp capping material (MTA).

After partial pulpotomy, no obliteration could be observed within any root canal system below the pulp capping material (Figs 1a and 1b, Figs 2a to 2d). However, in one tooth, obliteration of the pulp chamber – particularly in the area of the pulp horn close to the pulp capping material – was apparent (compare post-operative radiograph [Fig 1a] vs radiograph after 8 years [Fig 1b]). The radiographic assessment revealed no dentin-bridge formation in contact with the pulp capping material.

### Success Rate

**Fig 3:**
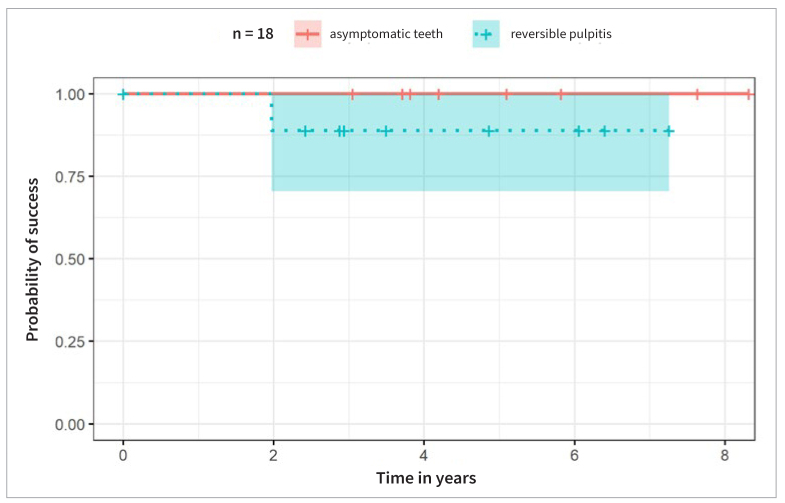
Kaplan-Meier-Plot with corresponding numbers at risk and cumulative number of events for each time point. The overall success (n = 18) is depicted for teeth with reversible pulpitis (n = 10, blue) and asymptomatic teeth (n = 8, red). ― asymptomatic teeth; -+- teeth with reversible pulpitis.

The success rates within the two groups (asymptomatic teeth vs reversible pulpitis) after partial pulpotomy according to strict clinical and radiographic criteria as mentioned above are presented as Kaplan-Meier curves (Fig 3). There was no statistically significant difference in the success rate between the groups (log-rank test: p = 0.3). At the 2-year follow-up, one patient, previously diagnosed with reversible pulpitis was diagnosed with a vertical root fracture. The tooth was thus classified as a failure. The estimated overall success rate at 4 years using the Kaplan-Meier method was 94.1% (95% CI: 0.84-1.00).

## Discussion

The present study’s estimated success rate of 94.1% at follow-up periods of 2-8 years (median 4 years) shows that partial pulpotomy after carious pulp exposure leads to high success rates even in the long term, at least in teeth with a low degree of inflammation of the pulp tissue. The only failure in the present study was due to a longitudinal fracture of a tooth, which is unlikely to be related to the treatment procedure of partial pulpotomy.

While some arguments have been made in the past against attempting any form of pulpotomy, stating that root canal treatment provides more predictable outcomes for patients, it is important to carefully consider the comparisons between partial pulpotomy, full pulpotomy, and root canal treatment. Partial pulpotomy is typically performed on asymptomatic teeth or those with reversible pulpitis, while full pulpotomy is reserved for cases of irreversible pulpitis. Given the ongoing debate surrounding the significance of this distinction, it is advisable to avoid direct comparisons between the two methods. Currently, there is no definitive consensus on this matter. In contrast to root canal treatment, pulpotomy (partial/full) and other VPT offer the advantage of preserving the sensory function of the pulp, and in young permanent teeth, root development can continue. Pulpotomy is less invasive compared to root canal treatment in terms of dentin structure loss during root canal preparation, and the risk of complications that could result in tooth loss is lower. Asgary et al^4^ documented the treatment time for four different VPTs and demonstrated that the more invasive the treatment procedure, the more time-consuming it is. When considering cost-effectiveness and tooth preservation, maintaining pulp vitality is preferable to root canal treatment, and any kind of endodontic complication should be avoided for both the preservation of tooth structure and the patient’s overall well-being.^14,38,39^

On the other hand, the various techniques of selective caries excavation – which aim to avoid pulpal exposure at all costs – must also be taken into account. A recently published systematic review has indicated that selective removal of carious tissue is more successful than nonselective removal.^6^ However, a closer examination of the four studies included in the meta-analysis raises concerns about the limited power of these studies. They possess partially retrospective study designs, employ outdated pulp capping materials, and have relatively short recall periods ranging between 1 to 3 years. Furthermore, it is important to note that nonselective removal and direct pulp capping should not be equated with partial pulpotomy, as the latter has demonstrated superior outcomes compared to direct pulp capping.^2,21^

The high success rate of our partial pulpotomies correlates with the results of other clinical studies^23,25^ and can be explained by several factors: firstly, none of the treated teeth had irreversible pulpitis, which was confirmed by the MMP-9 level measurements. The outcome of VPT is clearly associated with the inflammatory state of the pulp.^15^ The selection of the study teeth was very strict; teeth for which it was impossible to unequivocally assign a clear pulpal diagnosis were excluded right at the start. Secondly, a hydraulic calcium silicate cement (ProRoot MTA) was used for pulp dressing, which is associated with higher success rates in VPT procedures compared to the use of calcium hydroxide.^1,12,15^ Finally, the treatment protocol was as standardised as possible and all teeth were immediately restored by the direct placement of composite resin.

However, the results of the present study do not align with the results of a clinical study in which a success rate of only 11% after 5 years was determined after carious pulp exposure for teeth with asymptomatic pulp or reversible pulpitis.^8^ The significantly poorer success rates of that study^8^ may be due to the use of the calcium hydroxide-containing pulp-capping material Dycal (Dentsply Sirona; Konstanz, Germany) instead of pure calcium hydroxide paste, freshly prepared on site from calcium hydroxide and sterile saline^23,31^ or MTA,^31,34^ as used in other studies with high clinical success rates. Self-setting calcium hydroxide-containing materials such as Dycal or Life (Kerr; Orange, CA, USA) contain various other ingredients in addition to calcium hydroxide, such as sulfonamide (plasticiser) or butylene glycol disalicylate as setting activator.^16^ It is conceivable that the addition of these other components results in higher cytotoxicity^32,33^ and lower release of hydroxyl and calcium ions.^16,29^ Remarkable in this context are the results of the clinical study by Taha and Khazali,^42^ in which the success rates after partial pulpotomy after carious exposures – all teeth diagnosed with irreversible pulpitis – were still 85% after a follow-up period of 2 years when using MTA, and only 43% when using Dycal. In both animal experiments^11^ and clinical trials, the use of self-setting calcium hydroxide-containing materials has frequently resulted in low success rates after direct pulp capping.^3,7^

Another step of the procedure in the study by Bjørndal et al^8^ which may have lowered the success rates was the delayed final restoration after VPT. In that study, all cavities were temporarily restored with glass-ionomer cement for a period of 1 month and only then restored with composite resin.^8,9^ In the long term, even a small delay of the final restoration after VPT is associated with a reduced outcome.^7,10,26^ Furthermore, the glass-ionomer cement used by Bjørndal et al^8^ for temporary restorations is also considered unfavourable when applied close to the pulp^28^ and is associated with a reduced success rate after VPT.^7^ For this reason, the immediate restoration of all teeth using composite resin was performed in the present study, in accordance with the recommendations of the American Association of Endodontists (AAE).^1^

If radiographic and clinical examination alone are used for pulp diagnostics, the inflammatory state of the pulp may be misdiagnosed.^18,22^ Following the suggestion by Wolters et al^43^ to differentiate between more stages of pulpal inflammation than reversible and irreversible pulpitis based on clinical and radiographic findings, MMP-9 measurements could help to determine the exact grade of pulpal inflammation. This may allow a more accurate assessment of the prognosis after partial pulpotomy and pulpotomy.^40^ Measurement of MMP-9 levels could be a valuable tool to determine the correct diagnosis and consequently the appropriate therapy.^5,40^ In our study, MMP-9 levels showed a statistically significant difference between the two diagnosis-related groups (p = 0.006) and therefore confirmed the assignment of the teeth to the two groups. The measured MMP-9 levels in asymptomatic teeth were found to be half that of teeth diagnosed with reversible pulpitis. Moreover, the MMP-9 measurements served to accurately identify teeth with irreversibly inflamed pulp, preventing misdiagnosis. A systematic review on inflammatory mediators of pulpal inflammation points to the likelihood that measurements of MMP-9 levels can distinguish between reversible and irreversible pulpitis.^45^ However, the assessment of MMP-9 levels is still time-consuming and cost-intensive, and most pulp diagnoses can be accurately determined by accurate clinical and radiographic assessments.^36^

An essential role in evaluating the degree of inflammation of the pulp is the bleeding duration after partial pulpotomy, which was therefore an important diagnostic criterion defined before the start of the study.^27^ Thus, it is not surprising that the difference in bleeding duration after partial pulpotomy between the two groups was statistically significant (p = 0.009). Furthermore, the size of the exposed pulp after caries removal was statistically significantly larger in teeth with reversible pulpitis than in asymptomatic teeth (p = 0.043). This corresponds with the assumption that teeth with a larger carious lesion are more likely to develop an increased pulpal inflammation.^37^ Increased degrees of pulpal inflammation are associated with reduced success rates following VPT.^13,40^

In contrast to other treatment protocols, we used chlorhexidine solution after partial pulpotomy, which has the advantage of not falsifying bleeding duration compared to sodium hypochlorite, as discussed by Ballal et al.^5^ A longer bleeding duration can be indicative of a greater degree of inflammation of the pulp, which should not be masked by hemostatic agents. Moreover, chlorhexidine seems to have a positive influence on the dentin bond-strength stability by inhibiting MMPs.^17,19,24^

None of the root canals in this study cohort were obliterated below the pulp capping material (Figs 1a and 1b, Figs 2a to 2d). Only one tooth showed an obliteration of the coronal part of the pulp chamber and a minor diffuse calcification within the coronal third of the root canal, but not obliteration of the entire root canal system, even after a follow-up period of 8 years (Fig 1a vs 1b). Obliterations may potentially hinder root canal treatment the respective tooth that may become necessary at a later stage.^23,41^ Therefore, long-term absence of obliteration of the root canal system after VPT is desirable.

A drawback of the present pilot study is the small number of cases. Since MMP-9 measurements are very time-consuming and costly, they could only initially be performed over a limited period of time. Nevertheless, even with a small number of cases, important information can be drawn from the evaluated long-term results.

## Conclusions

The present pilot study’s results indicate that with the treatment option of partial pulpotomy in permanent teeth after carious pulp exposure, high success rates can be expected – even in the long term – if the pulp tissue shows no or reversible signs of inflammation. Further clinical studies with larger cohorts are necessary to confirm these results.
